# The Significance of Transarterial Chemo(Embolization) Combined With Tyrosine Kinase Inhibitors and Immune Checkpoint Inhibitors for Unresectable Hepatocellular Carcinoma in the Era of Systemic Therapy: A Systematic Review

**DOI:** 10.3389/fimmu.2022.913464

**Published:** 2022-05-23

**Authors:** Qiao Ke, Fuli Xin, Huipeng Fang, Yongyi Zeng, Lei Wang, Jingfeng Liu

**Affiliations:** ^1^ Department of Hepatopancreatobiliary Surgery, Mengchao Hepatobiliary Hospital of Fujian Medical University, Fuzhou, China; ^2^ College of Clinical Medicine for Oncology, Fujian Medical University, Fuzhou, China; ^3^ Department of Radiation Oncology, Fujian Cancer Hospital, Fujian Medical University Cancer Hospital, Fuzhou, China; ^4^ Department of Hepatopancreatobiliary Surgery, Fujian Cancer Hospital, Fujian Medical University Cancer Hospital, Fuzhou, China; ^5^ The United Innovation of Mengchao Hepatobiliary Technology Key Laboratory of Fujian Province, Mengchao Hepatobiliary Hospital of Fujian Medical University, Fuzhou, China

**Keywords:** hepatocellular carcinoma, transarterial chemotherapy, hepatic arterial infusion chemotherapy, tyrosine kinase inhibitors, immune checkpoint inhibitors, systematic review

## Abstract

**Background and Aims:**

Regardless of great progress in early detection of hepatocellular carcinoma (HCC), unresectable HCC (uHCC) still accounts for the majority of newly diagnosed HCC with poor prognosis. With the promising results of a double combination of transarterial chemo(embolization) and tyrosine kinase inhibitors (TKIs), and TKIs and immune checkpoint inhibitors (ICIs), a more aggressive strategy, a triple combination of transarterial chemo(embolization), TKIs, and ICIs has been tried in the recent years. Hence, we aimed to conduct a systematic review to verify the safety and efficacy of the triple therapy for uHCC.

**Methods:**

PubMed, MedLine, Embase, the Cochrane Library, and Web of Knowledge were used to screen the eligible studies evaluating the clinical efficacy and safety of triple therapy for patients with uHCC up to April 25th 2022, as well as Chinese databases. The endpoints were the complete response (CR), objective response rate (ORR), disease control rate (DCR), conversion rate, progression-free survival (PFS) rate, overall survival (OS) rate, and the incidence of adverse events (AEs).

**Results:**

A total of 15 studies were eligible with 741 patients receiving transarterial chemoembolization (TACE) or hepatic arterial infusion chemotherapy (HAIC) combined with TKIs and ICIs. The pooled rate and 95% confidence interval (CI) for CR, ORR, and DCR were 0.124 (0.069–0.190), 0.606 (0.528–0.682), and 0.885 (0.835–0.927). The pooled rates for PFS at 0.5 years and 1 year were 0.781 (0.688–0.862) and 0.387 (0.293–0.486), respectively. The pooled rates for OS at 1, 2, and 3 years were 0.690 (0.585–0.786), 0.212 (0.117–0.324), and 0.056 (0.028–0.091), respectively. In addition, the pooled rate and 95%CI for the conversion surgery was 0.359 (0.153–0.595). The subgroup analysis of control studies showed that triple therapy was superior to TACE+TKIs, TKIs+ICIs, and TKIs in CR, ORR, and DCR, conversion rate; PFS; and OS. No fatal AEs were reported, and the top three most common AEs were elevated ALT, elevated AST, and hypertension, as well as severe AEs (grading ≥3).

**Conclusion:**

With the current data, we concluded that the triple therapy of TACE/HAIC, TKIs, and ICIs would provide a clinical benefit for uHCC both in short- and long-term outcomes without increasing severe AEs, but the conclusion needs further validation.

**Systematic Review Registration:**

http://www.crd.york.ac.uk/PROSPERO/, Review registry: CRD42022321970.

## Introduction

Primary liver cancer is the sixth most common cancer worldwide with approximately 906,000 newly diagnosed patients per year and more than 90% patients having hepatocellular carcinoma (HCC) (1). The prognosis of HCC patients remains far from satisfactory with the median overall survival (OS) of 25–30 months (2, 3). Radical surgery is still the most cost-effective curative treatment for HCC patients, but the majority have lost the chance of surgery, so called “unresectable” HCC (uHCC), mainly due to the absence of symptoms in the early stage of HCC (4–6).

There are no guidelines or consensus on the management of uHCC patients up to now (4–6) because this population is too heterogeneous. For uHCC patients at intermediate stage according to the Barcelona Clinic Liver Cancer stage (BCLC) (6), transarterial chemoembolization (TACE), as a classical modality of transarterial chemo(embolization), is strongly recommended with the overall objective response rate (ORR) beyond 50% (7, 8). In the recent years, another modality of transarterial chemo(embolization), hepatic arterial infusion chemotherapy (HAIC), has been identified as non-inferior to TACE in the management of HCC, and particularly, the advantage of HAIC over TACE has been verified among those with macrovascular invasion (9, 10). Nonetheless, the prognosis of patients receiving TACE/HAIC remains poor with the median progression-free survival (PFS) of 2.8–9.6 months and most of the patients will get resistant after repeated TACE (9, 11). For advanced uHCC patients including those with extrahepatic metastasis, systemic therapy is the preferred strategy (12, 13). With the advent of the novel tyrosine kinase inhibitors (TKIs) and immune checkpoint inhibitors (ICIs), sorafenib is not the only option for advanced HCC (14, 15). In addition, the double-combination modality of systemic therapy, such as atezolizumab and bevacizumab (15), lenvatinib and pembrolizumab (16), durvalumab and tremelimumab (17), apatinib and camrelizumab (18), and novolumab and ipimumab (19), have exhibited promising results with manageable toxicity. However, the objective response rate (ORR) of systemic therapy is still poor, and the time to response might be too long.

The combination of locoregional and systemic treatments is another option for uHCC (3). In theory, locoregional treatment, such as TACE or HAIC, is efficient to achieve satisfactory local control (LC); however, it has not always translated into a long-term survival benefit. On the other hand, systemic therapy is the key to improve the long-term prognosis, but unsatisfactory LC will impair the long-term survival advantage. Preclinical studies have identified the synergistic effect of TACE/HAIC and systemic therapy (20, 21), which was also confirmed in practice of the combination of TACE and sorafenib (22), TACE and lenvatinib (23), HAIC and sorafenib (24), and HAIC plus lenvatinib and toripalimab (25), but it is not the end.

With the publication of the IMbrave 150 trial (15), HCC has entered the era of molecular and immune therapy. It is surprising that approximately 40% patients were found to receive previous TACE before randomization in the IMbrave 150 trial, which shed light on a more aggressive modality, the triple therapy of TACE/HAIC, TKIs, and ICIs. Furthermore, the current strategy is far from enough to satisfy the increasing demands of “conversion therapy” for uHCC. In the past 2 years, the triple therapy of TACE/HAIC, TKIs, and ICIs has been tried with encouraging results (20, 25, 26), but most of the studies were retrospective with a small sample size. Therefore, in this study, we comprehensively reviewed all the literature on the triple therapy of TACE/HAIC, TKIs, and ICIs and aimed to provide substantial clues for the subsequent studies.

## Material and Method

This systematic review was conducted according to the preferred Reporting Items for Systematic Reviews and Meta-Analyses (PRISMA) guideline, which was also registered at http://www.crd.york.ac.uk/PROSPERO/ (Review registry 321970)

### Literature Searching

Using PubMed, MedLine, Embase, the Cochrane Library, and Web of Science, all literature was searched on the triple combination of TACE/HAIC, TKIs, and ICIs for uHCC. The keywords included “primary liver cancer” or “liver tumor” or “hepatocellular carcinoma” or “HCC”, and “transcatheter arterial chemoembolization” or “transarterial chemoembolization” or “hepatic arterial infusion chemotherapy” or “chemotherapy” or “TACE” or “HAIC”, and “tyrosine kinase inhibitors” or “TKIS” and “immune check point inhibitors” or “ICIs” or “programmed cell death protein 1” or “programmed cell death ligand 1” or “PD-1” or “PD-L1” or “B7-H1.” The literature searching began in December 2021, and the last searching was April 25th 2022. Considering that TACE or HAIC is preferred in China, the Chinese database of China National Knowledge Infrastructure(CNKI) and Wanfang were also used to identify the eligible studies.

### Selection Criteria

The inclusion criteria were as follows: i) patients diagnosed as HCC by image or biopsy, ii) unresectable after Multi-Disciplinary Team (MDT), and iii) received the triple combination modalities of TACE/HAIC, TKIs, and ICIs, regardless of sequence; iv) endpoints must consist at least one of the following items: complete response (CR), ORR, disease control rate (DCR), PFS, OS, conversion rate, and adverse events (AEs).

The exclusion criteria were as follows: i) combined with other treatment such as ablation or radiation, ii) duplicate report derived from the same cohort, iii) protocol, case reports or reviews, and iv) data unavailable.

### Data Acquisition

According to the predefined forms, the information of the eligible studies include the surname of the first author, year of publication, design of the study, and study period. In addition, baseline characteristics in each study (sample size, age, sex, hepatitis B virus infection, Child–Pugh grade, AFP level, tumor number, tumor size, macrovascular invasion, extrahepatic metastasis, BCLC stage, mean PFS, mean OS and regimen of TACE/HAIC, TKIs, and ICIs) were extracted directly by two independent researchers (QK and FX). Endpoints including the CR, ORR, DCR, PFS, OS, conversion rate, and adverse events were obtained directly from the main text or supplementary files and were then cross-validated between the researchers. In case of any discrepancy, an MDT discussion including at least one senior doctor was introduced to reach the final decision. Of note, tumor responses were determined by the modified response evaluation criteria in solid tumors (mRECIST) or RECIST in this study, but we would choose the results evaluated by mRECIST if they were also evaluated by RECIST in each included study.

### Quality Assessment

Considering that all the included studies were retrospective, the quality of each eligible study was assessed by the modified Newcastle–Ottawa Scale (NOS) (27). Briefly, the risk of bias was graphically presented as a proportion of all included studies. Evaluating elements included the following: i) whether the study reported a definite definition of the objective; ii) whether a clear triple combination of TACE/HAIC, TKIs, and ICIs was offered (including the technique, regimen, and course of TACE/HAIC and dosage and courses of TKIs and ICIs); iii) whether the criteria of response assessment was provided (i.e., RECIST or mRECIST); and iv) whether there was a clear definition of outcomes including THE CR, ORR, and DCR.

### Statistical Analysis

A meta-analysis of the pooled rate was conducted using Rstudio and R (4.1.2); while a comparison analysis between two groups was conducted using RevMan Version 5.3. The pooled rate for the CR, ORR, and DCR and rates of PFS and OS at different time-points was used as an effect size with 95% confidence interval (CI). The χ2 test and *I* ^2^ statistics were used to evaluate the heterogeneity among the included studies. If P>0.10 and *I* ^2^<50%, there was no apparent heterogeneity, and the fixed-effect model was used to estimate the effect size; otherwise, the random-effect model was used (28, 29). Sensitivity analysis was carried out by removing each of the included studies one by one to determine the reliability of the results. Subgroup analyses were also conducted to decrease the heterogeneity among the included studies. Publication bias was determined using the funnel plot with Egger’s and Begg’s tests. In this study, a P-value <0.05 was considered significant.

## Results

### Searching Results

**Figure 1:**
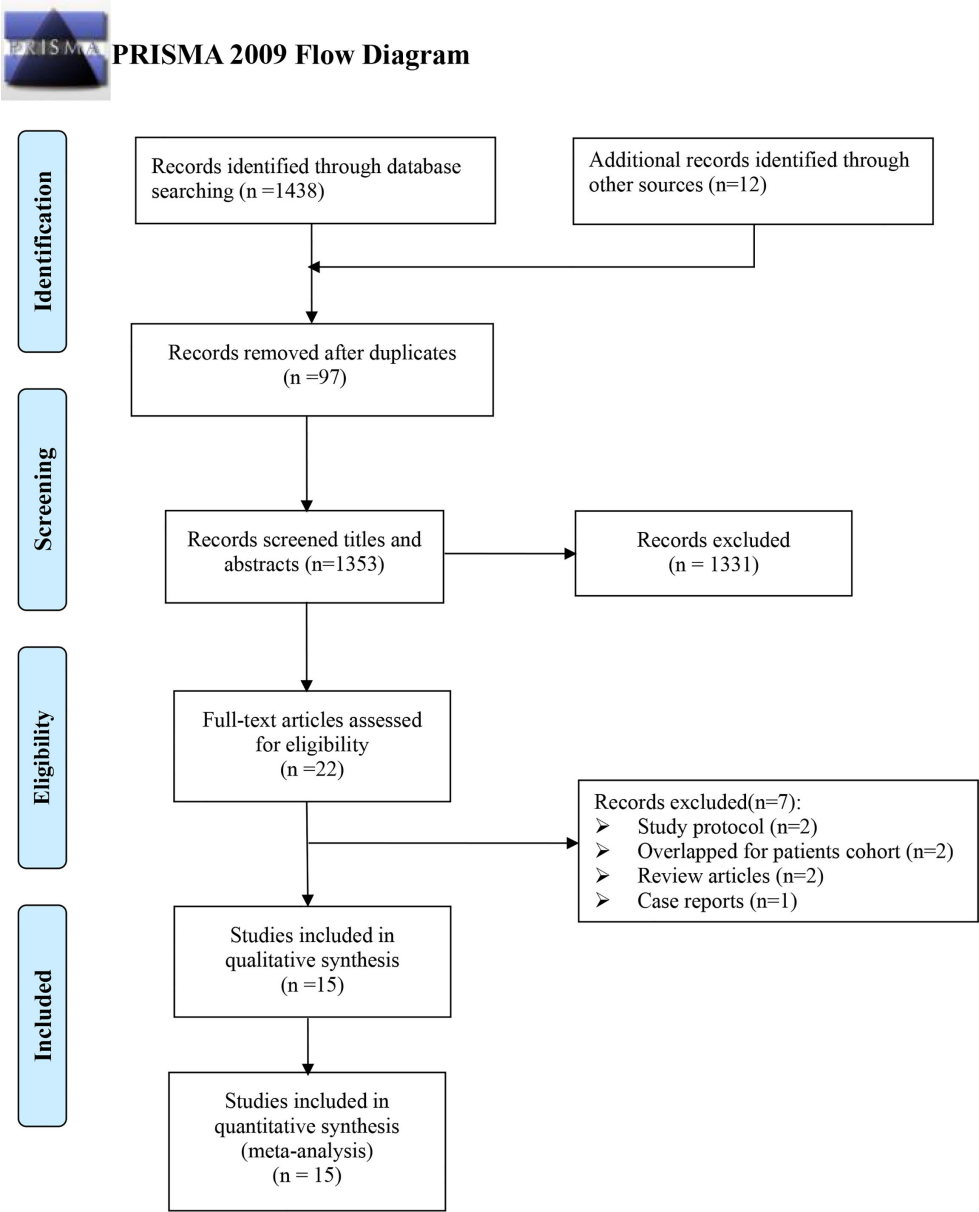
Flow diagram for study selection.

Initially, 1,450 records were identified using an electronic database, including 1,223 written in English and 227 in Chinese, respectively. A total of 97 were excluded by duplicating, 1,353 by screening titles and abstracts, and another 22 more by reading the full text. Finally, 15 records were assessed to be eligible for this meta-analysis (21, 25, 30–42) (**Figure 1**).

All of the included studies came from China, and all the studies were retrospective, three of which were multi-centered (25, 30, 34). The baseline characteristics in each study were depicted in **Table 1**, as well as the results of quality assessment. Of note, the baseline characteristics of the control arm in the comparing cohort studies were also depicted in **Table 1**.

**Table 1:** Baseline characteristics and quality assessment of included studies.

Study	Design	Treatment	Patients	Age	Sex	HBV	Child-pugh	AFP (ng/ml)	Tumor number	Tumor size	MVI	Extrahepatic metastasis	BCLC stage	Mean PFS	Mean OS	Quality
				year	M/F	Positive/Negative	A/B	<400/≥400	S/M	cm	Yes/No	Yes/No	A/B/C	month	month	
Wu 2021 (30)	R multi-center	TACE+Len+ICIs	62	57(23-75)	56/6	57/5	NA	30/32	25/37	31/31 (<10/≥10)	34/28	6/56	6/21/63	Not reached	Not reached	H
Chen 2021 (21)	R single center	TACE+Len+ pembrolizumab	70	58(36-69)	37/33	38/32	NA	25/45	NA	NA	NA	48/22	0/47/23	9.2	18.1	H
		TACE+Len	72	57(35-68)	38/34	44/28	NA	28/44	NA	NA	NA	52/20	0/45/27	5.5	14.1	
Liu 2021 (31)	R single center	TACE+Len+ camrelizumab	22	57.5 ± 9.9	17/5	15/7	16/6	15/7	NA	NA	11/11	NA	0/12/10	11.4	24.0	M
Cao 2021 (32)	R single center	TACE+Len+ sintilimab	52	40/12(65)	45/7	47/5	46/6	34/18	NA	NA	19/33	21/31	0/13/29	13.3	23.6	H
Zheng 2021 (33)	R single center	TACE+Sor+ICIs	22	10/12(55)	19/3	17/5	13/9	7/15	7/15	8/14 (<5/≥5)	7/15	7/15	0/11/11	16.3	23.3	H
		TACE+Sor	29	18/11(55)	24/5	27/2	18/11	8/21	9/20	15/14	8/21	13/16	0/14/15	7.3	13.8	
Chen 2021 (34)	R multi-center	HAIC+Len+ pembrolizumab	84	52(42-67)	72/12	45/39	71/13	3984 (82-49,534)	NA	NA	49/35	20/64	0/22/62	10.9	17.7	H
		Len+ pembrolizumab	86	53(43-69)	71/15	48/38	75/11	4022 (79-51,462)	NA	NA	55/31	24/62	0/21/65	6.8	12.6	
He 2021 (25)	R multi-center	HAIC+Len+ Pembrolizumab	71	40/31 (≤50/>50)	59/12	62/9	NA	26/45	NA	26/45 (<10/≥10)	55/16	16/55	NA	11.1	Not reached	H
		levatinib	86	42/44 (≤50/>50)	77/9	78/8	NA	31/55	NA	40/46 (<10/≥10)	62/24	25/61	NA	5.1	11	
Mei 2021 (35)	R single center	HAIC+Len+ ICIs	45	49.1 ± 10.6	38/7	37/8	44/1	4106 (72.8-121,000)	36/9	11.2 ± 3.9	36/9	15/30	0/5/40	8.8	15.9	H
		Len+ICIs	25	50.1 ± 12.3	18/7	19/6	22/3	767.6 (23.3-21,940.5)	20/5	10.9 ± 4.2	18/7	13/12	0/3/22	5.4	8.6	
Zhang 2021 (36)	R single center	HAIC+TKIs+ ICIs	25	62.0(49-78)	19/6	22/3	22/3	11/14	NA	NA	23/0	NA	0/0/25	Not reached	Not reached	M
Liu 2021 (37)	R single center	HAIC+TKIs+ ICIs	27	59.2 ± 1.4	26/1	25/2	22/5	12/17	12/15	7.7 ± 0.7	20/7	8/19	0/0/27	10.6	NA	M
Yang 2022 (38)	R single center	TACE+TKIs +ICIs	31	57.5 ± 9.4	6/25	26/5	27/4	23/8	12/19 (≤3/>3)	NA	NA	NA	2/18/11	8.5	NA	M
Ju 2022 (39)	R single center	TACE+ apatinib+ camrelizumab	56	52(26-75)	46/10	48/8	43/13	21/35 (<200/≥200)	9/47	9.7 ± 4.9	NA	NA	0/13/43	NA	24.8	H
Cai 2022 (40)	R single center	TACE+Len+ICIs	41	51.9 ± 10.3	37/4	35/5	37/4	20/21	18/23 (≤3/>3)	12.3 ± 4.8	15/26	17/24	NA	7.3	16.9	H
		TACE+Len	40	54.6 ± 11.0	33/7	35/6	33/7	18/22	21/19 (≤3/>3)	13.6 ± 5.1	18/22	19/21	NA	4.0	12.1	
Teng 2022 (41)	R single center	TACE+Len+ICIs	53	56.9(37-75)	45/8	45/8	34/19	35/18	NA	NA	25/28	42/11	0/23/30	8.5	Not reached	M
Ju 2022 (42)	R single center	TACE+ apatinib+ camrelizumab	80	52(46-62)	66/14	65/15	58/22	28/52 (<200/≥200)	13/67	9.7 ± 4.7	47/33	44/36	0/13/67	15.7	22.1	H

TACE, transarterial chemoembolization; HAIC, hepatic artery infusion chemotherapy; TKIs, tyrosine kinase inhibitors; ICIs, immune checkpoint inhibitors; Len, lenvatinib; Sor, sorafenib; R, retrospective; M, male; F, female; HBV, hepatitis B virus; PS, performance status; S, single; M, multiple; MVI, macrovascular invasion; BCLC, Barcelona Clinic Liver Cancer Stage; OS, overall survival; PFS, progression-free survival; H, high; M, medium; NA, not available.

Considering that there was no consensus on the triple combination of TACE/HAIC, TKIs, and ICIs, the scheme in each study was a little different from each other. **Table 2** exhibited the detailed information on the treatment scheme, including the technique of TACE/HAIC, drug regimens, and the sequence of local and systemic therapy.

**Table 2:** Detailed scheme of triple therapy included studies.

Studies	TACE/HAIC	TKIs	ICIs
Wu 2021 (30)	TACE: performed every 4–6 weeks if there was obvious hepatic arterial blood supply to HCC according to contrast enhanced abdominal CT or MRI.	Lenvatinib: 8 mg for body weight <60 kg or 12 mg for body weight ≥60 kg) Oral, once a day Treatment was stopped for 3 days before and after TACE	Sintilimab 200 mg, tislelizumab 200 mg, camrelizumab 200 mg, toripalimab 240 mg, or pembrolizumab 200 mg intravenous injection, once every 3 weeks Treatment was stopped for 3 days before and after TACE
Chen 2021 (21)	TACE: performed after the combination treatment of TKIs and PD-1, and was repeated if the lesion reduction was less than 50% of the baseline.	Lenvatinib: 8 mg, regardless of body weight Oral, once a day	Pembrolizumab 200 mg Intravenous injection, once every 3 weeks
Liu 2021 (31)	TACE: raltitrexed diluent (4 mg) + oxaliplatin (100 mg) +lipiodol (10-20 ml) + pirarubicin (20 mg) + gelfoam particles Procedure only approximately 1–3 times based on imaging examination findings	Lenvatinib: 8 mg for body weight <60 kg or 12 mg for body weight ≥60 kg) Oral, once a day	Camrelizumab 200 mg Intravenous injection, once every 3 weeks
Cao 2021 (32)	TACE: oxaliplatin (75 mg/m^2^) + iodized oil mixed with epirubicin (30-50 mg/m^2^) The TACE procedure was repeated 4-6 weeks later.	Lenvatinib: 8 mg for body weight <60 kg or 12 mg for body weight ≥60 kg), and was initiated at 2 weeks pre-TACE Oral, once a day	Sintilimab 200 mg Intravenous injection, once every 3 weeks Initiated at day 1 after the TACE procedure
Zheng 2021 (33)	TACE: oxaliplatin (100-150 mg) + 5-fluorouracil (500-750 mg)+ hyper-liquefying iodide oil (10-30 ml) + epirubicin (10–20 mg) + gelatin sponge particles Repeated TACE would be recommended once the lipiodol deposition shrank and residual lesions occurred, indicating viable lesions or intrahepatic recurrence by contrast-enhanced MRI within 6 weeks after TACE therapy.	Sorafenib: 400 mg and was initiated within 2 weeks post-TACE Oral, twice a day	Nivolumab or pembrolizumab (3 mg/kg) Intravenous injection, once every 3 weeks
Chen 2021 (34)	HAIC: 85 mg/m^2^ oxaliplatin from hour 0 to 2 on day 1; 400 mg/m^2^ fluorouracil bolus at hour 3 and 2,400 mg/m^2^ fluorouracil over 46 h on days 1 and 2; and 400 mg/m^2^ leucovorin from hour 2 to 3 on day 1 Once every 3 weeks	Lenvatinib: 8 mg for body weight <60 kg or 12 mg for body weight ≥60 kg) Oral, once a day	Pembrolizumab intravenously once every 3 weeks
He 2021 (25)	HAIC: oxaliplatin 85 mg/m^2^ from hour 0 to 2 on day 1; leucovorin 400 mg/m^2^ from hour 2 to 3 on day 1; 5-fluorouracil 400 mg/m^2^ bolus at hour 3; and 2,400 mg/m^2^ over 46 h on days 1 and 2.	Lenvatinib: 8 mg for body weight <60 kg or 12 mg for body weight ≥60 kg) orally once daily	Toripalimab 240 mg intravenous injection, once every 3 weeks Initiated at 0–1 day prior to HAIC
Mei 2021 (35)	HAIC: 85 or 135 mg/m^2^ oxaliplatin, 400 mg/m^2^ leucovorin, and 400 mg/m^2^ fluorouracil on the first day; and 2,400 mg/m^2^ fluorouracil over 46 h.	Lenvatinib: 8 mg for body weight <60 kg or 12 mg for body weight ≥60 kg) orally once daily Treatment was initiated within 3 days before or after the start of HAIC	Sintilimab 200 mg, toripalimab 240 mg, or pembrolizumab 200 mg, pembrolizumab 200 mg, nivolumab 100 mg Treatment was initiated within 3 days before or after the start of HAIC
Zhang 2021 (36)	HAIC: oxaliplatin 85 mg/m^2^ as a 2 h infusion, calcium folinate 400 mg/m^2^ as a 2–3 h infusion, and fluorouracil 400 mg/m^2^ as a bolus injection, followed by fluorouracil 1,200 mg/m^2^ administered over 23 h on day 1 Every 4–8 weeks	Apatinib 250 mg/day, lenvatinib 8 mg/day, or sorafenib 400 mg twice daily Oral	Camrelizumab 200 mg or sintilimab 200 mg, intravenous injection, once every 3 weeks
Liu 2021 (37)	HAIC: FOLFOX (oxaliplatin, 60–75 mg/m^2^ HAIC for 0–4 h; (Child–Pugh A, 75 mg/m^2^; and Child–Pugh B7, 60 mg/m^2^), 5-fluorouracil, 1-1.5 g/m^2^ HAIC for 4–24 h (Child–Pugh A, 1.5 g/m^2^; and Child–Pugh B, 1 g/m^2^) and leucovorin (200 mg, intravenous infusion for 2 h before 5-Fu) was used. HAIC was repeated every 4–6 weeks until the intrahepatic lesions achieved CR, disease progression, or until the toxicity was unacceptable	Lenvatinib: 8 mg per day oral Sorafenib: 200 mg oral, twice daily; 400 mg was administered orally twice daily if drug tolerance was acceptable. If patients had received sorafenib or lenvatinib before the study, regorafenib or apatinib was given. Regorafenib: Approximately 80 mg was administered orally once daily; 120 mg was administered orally once daily if drug tolerance was acceptable. Apatinib: 250 mg was administered orally once daily for 28 days as a treatment cycle.	Camrelizumab (200 mg/3 weeks), sintilimab (200 mg/3 weeks), toripalimab (240 mg/3 weeks), and nivolumab (3 mg/kg every 2 weeks).
Yang 2022 (38)	TACE:lipiodol (5-20 ml)+pirarubicin (10-20 mg) + gelatin sponge or polyvinyl alcohol particles (300-500 mm, if necessary)	Lenvatinib: 8 mg for body weight <60 kg or 12 mg for body weight ≥60 kg), oral, once a day Sorafenib: 400 mg, oral, twice a day Treatment was suspended during the TACE procedure and resumed after TACE	Camrelizumab 200 mg, intravenous injection, once every 3 weeks Treatment was suspended during the TACE procedure and resumed after TACE
Ju 2022 (39)	TACE: the modality includes the following: 1) different diameter drug-eluting beads loaded with 60 mg doxorubicin; and 2) the iodine oil–Doxorubicin (DOX) emulsion, a water-in-oil type of chemoembolization, which was prepared by using doxorubicin mixed with lipiodol	Apatinib: 250 mg, oral, once a day Treatment was suspended 3 days before the following the TACE procedure	Camrelizumab 200 mg, intravenous injection, once every 3 weeks
Cai 2022 (40)	cTACE: lipiodol (5-20 ml)+pirarubicin (20-60 mg) + polyvinyl alcohol particles (90-500 mm) DEB-TACE: CalliSpheres or DC bead (100-300 μm), and one vial of the beads was loaded with 60 mg pirarubicin	Lenvatinib: 8 mg for body weight <60 kg or 12 mg for body weight ≥60 kg) and was initiated within 7 days after the first TACE Oral, once a day	Sintilimab, tislelizumab, or camrelizumab 200 mg Intravenous injection, once every 3 weeks Initiated within 7 days after the first TACE
Teng 2022 (41)	TACE: lipiodol (5-20 ml)+ epirubicin (50 mg) + embosphere microspheres (300-500 mm)	Lenvatinib: 8 mg for body weight <60 kg or 12 mg for body weight ≥60 kg) and was initiated within 1-2 weeks before TACE Oral, once a day	Camrelizumab 200 mg or sintilimab 200 mg, intravenous injection, once every 3 weeks Initiated within 1 week after TACE
Ju 2022 (42)	cTACE: lipiodol+doxorubicin+absorbable gelatin sponge particles (350-560 mm) DEB-TACE: CalliSpheres of different diameters loaded with 60 mg of doxorubicin	Apatinib: 250 mg, oral, once a day and was initiated within 1 week after TACE Treatment was suspended 3 days before the next TACE	Camrelizumab 200 mg, intravenous injection, once every 3 weeks Initiated within 1 week after TACE

cTACE, conventional transarterial chemoembolization; DEB-TACE, drug-eluting beads transarterial chemoembolization; HAIC, hepatic artery infusion chemotherapy; TKIs, tyrosine kinase inhibitors; ICIs, immune checkpoint inhibitors.

### Endpoints

**Figure 2:**
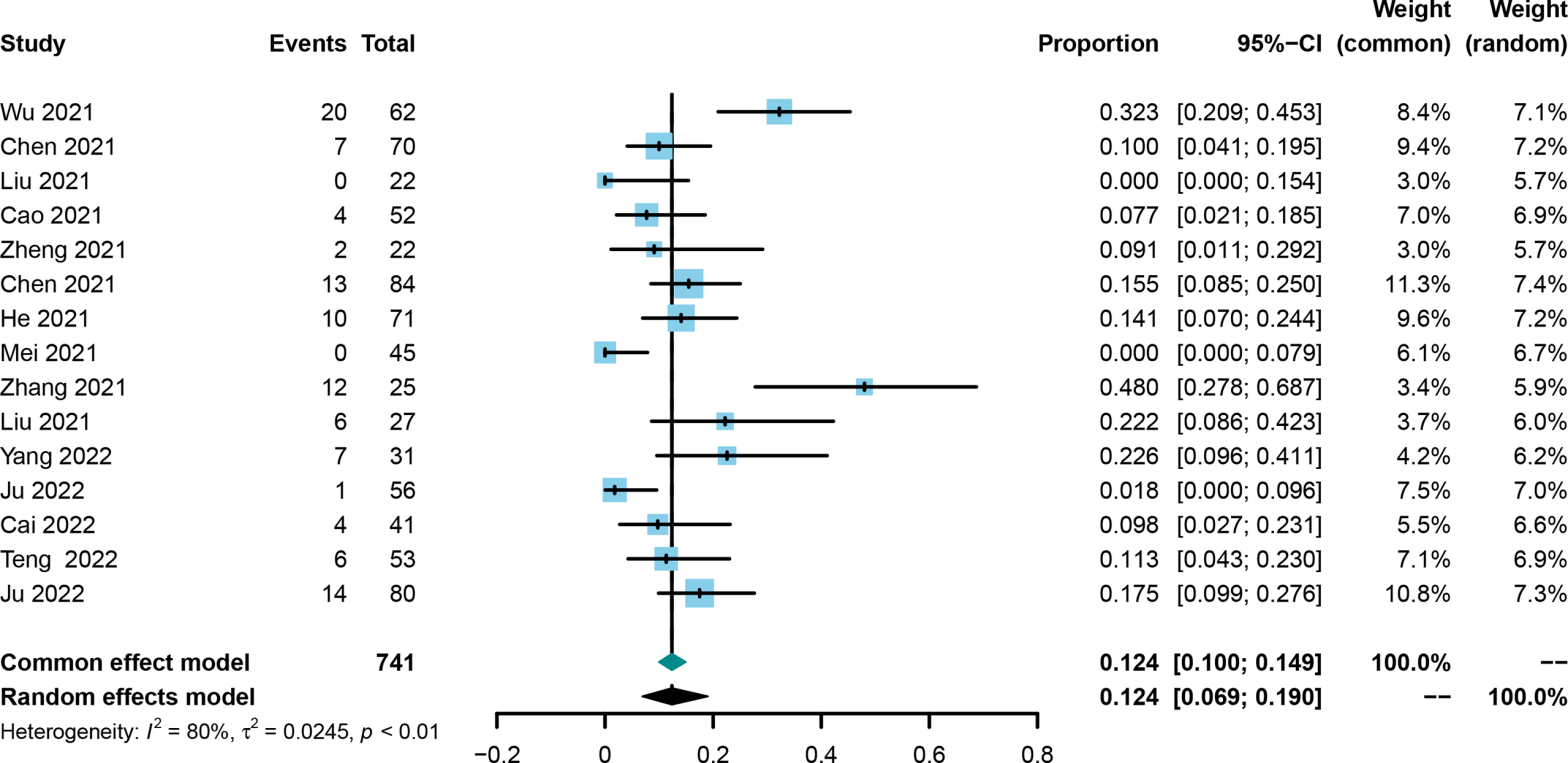
Forest plot of the pooled complete response.

The CR was evaluated in all included trials (21, 25, 30–42), and the corresponding rates ranged from 0% to 48.0%. Using the random effect model, the pooled rate and 95%CI for CR was 0.124 (0.069-0.190, **Figure 2**). Asymmetry was not observed by the funnel plot (**Supplementary Figure 1**) with the Egger’s test of 0.9846 and Begg’s test of 0.7662. Sensitivity analysis showed that the results did not change greatly after removing any included single study (**Supplementary Figure 2**).

**Figure 3:**
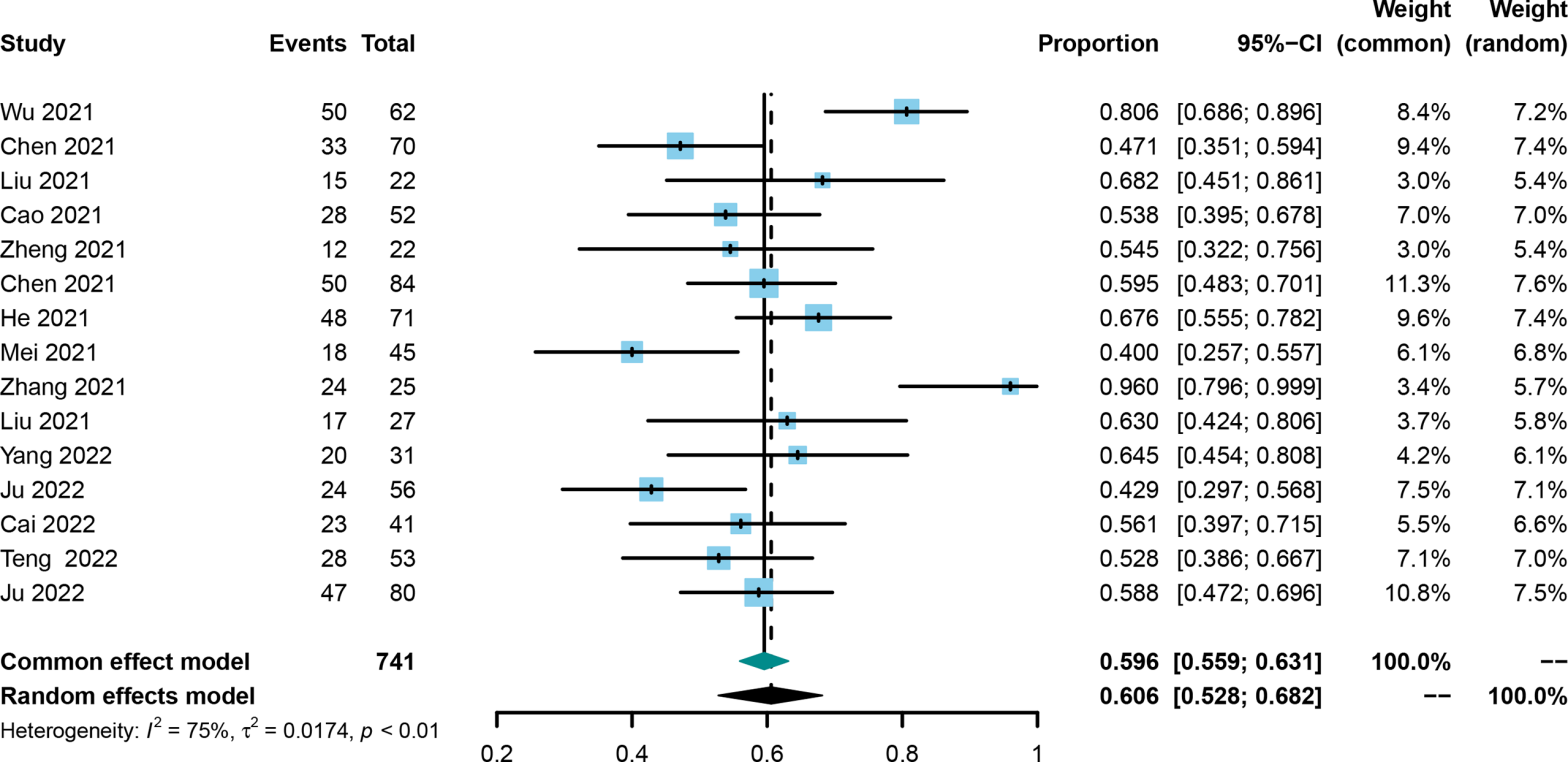
Forest plot of the pooled objective response rate.

ORR was evaluated in all included trials (21, 25, 30–42), and the pooled ORR and 95% CI was 0.606 (0.528-0.682, **Figure 3**) using the random effect model. No publication bias was identified using the funnel plot (**Supplementary Figure 3**) and Egger’s and Begg’s tests (0.4223 and 0.4879, respectively), and the result was not influenced by any one of the included studies (**Supplementary Figure 4**).

**Figure 4:**
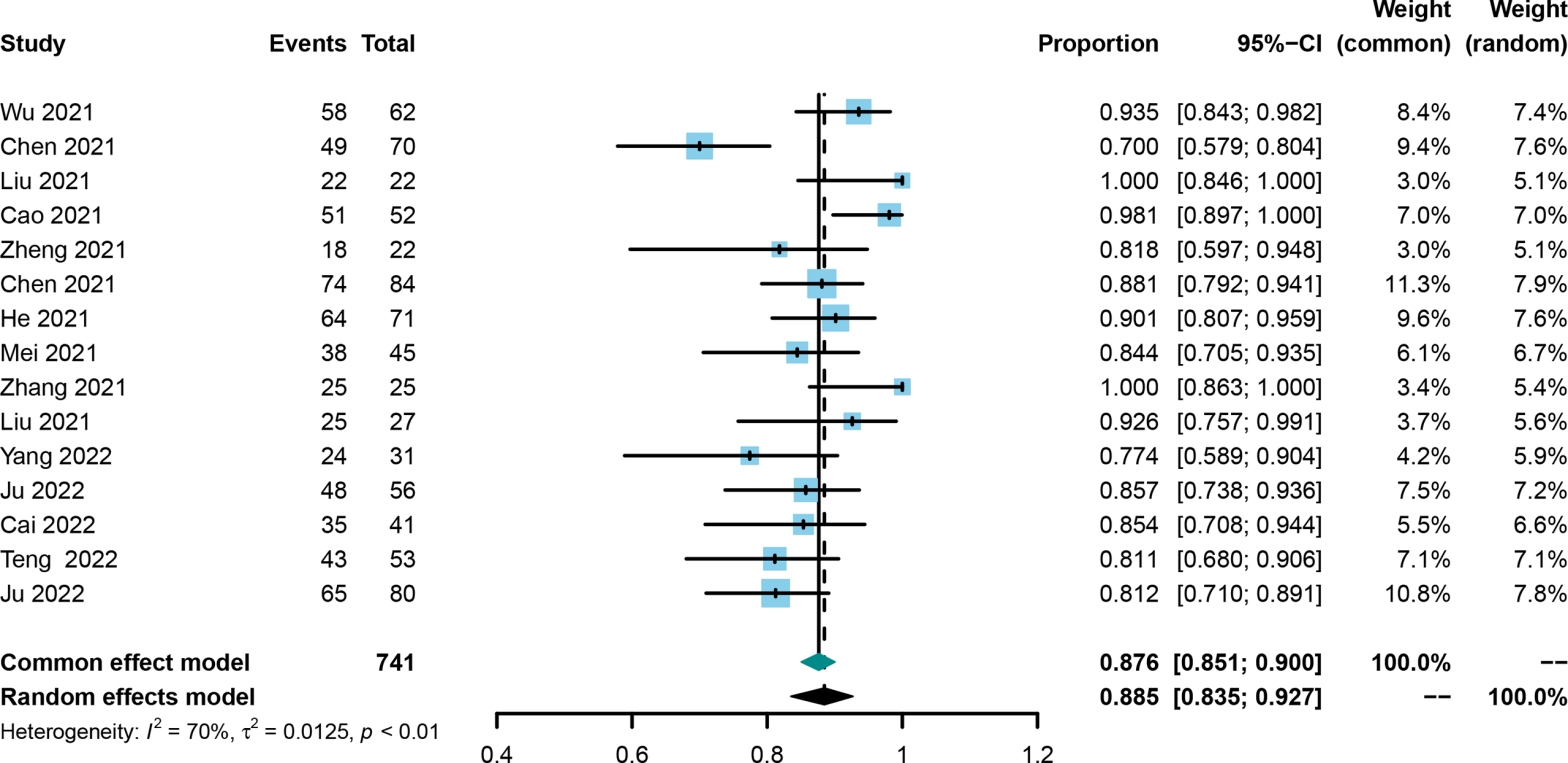
Forest plot of the pooled disease control rate.

The DCR was also evaluated in all included trials (21, 25, 30–42), and the corresponding rates ranged from 70.0% to 100%. Using the random effect model, the pooled DCR was 0.885 with the 95%CI of 0.835–0.927 (**Figure 4**). Publication bias was not observed among the included studies (**Supplementary Figure 5**), and the stability of the result was confirmed by sensitivity analysis (**Supplementary Figure 6**).

**Figure 5:**
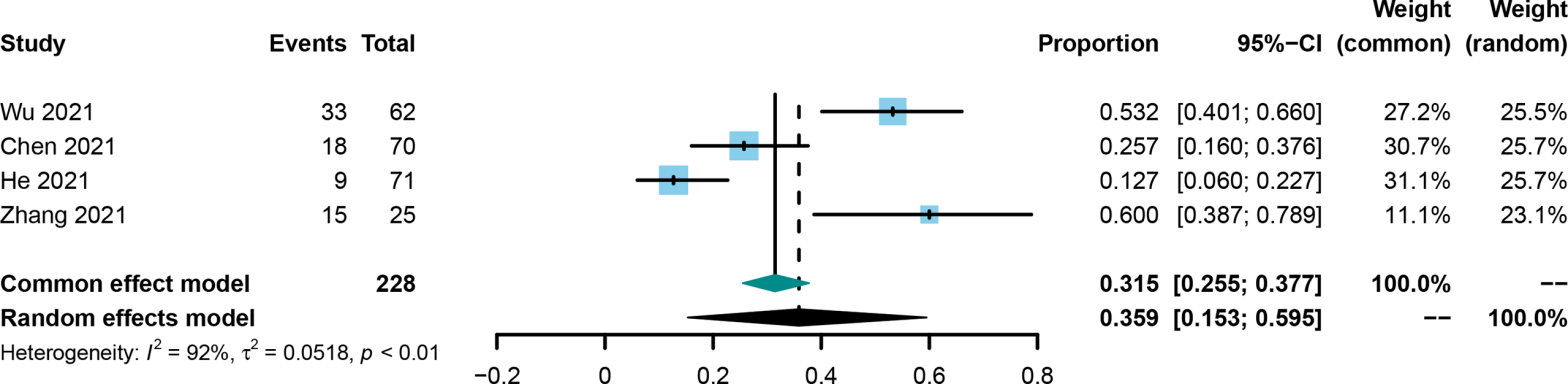
Forest plot of the pooled surgical conversion rate.

The conversion rate was evaluated in four included studies (21, 25, 30, 36). The conversion rate in the included studies was from 12.7% to 60.0%, and the pooled rate and 95%CI was 0.359 (0.153-0.595, **Figure 5**) using the random effect model.

PFS at 0.5, 1, 1.5, and 2 years were evaluated in 13 (21, 25, 31–38, 40–42), 13 (21, 25, 31–38, 40–42), 11 (21, 25, 31–37, 41, 42), and 5 studies (21, 32, 34, 37, 42), and the pooled rates and 95%CI were 0.781 (0.688-0.862), 0.387 (0.293-0.486), 0.117 (0.076-0.165), and 0.069 (0.005-0.182), respectively (**Table 3**). OS at 0.5, 1, 1.5, 2, and 3 years were evaluated in 12 (21, 25, 31–36, 39–42), 12 (21, 31–34, 39–42), 12 (21, 31–34, 39–42), 9 (21, 31–34, 39–42), and 5 studies (21, 31–33, 42), and the pooled rates and 95%CI were 0.943 (0.902-0.975), 0.690 (0.585-0.786), 0.385 (0.246-0.533), 0.212 (0.117-0.324), and 0.056 (0.028-0.091), respectively (**Table 3**).

**Table 3:** Progression-free survival and overall survival of included studies.

Endpoints		Included studies	Participants	Effect model	Proportion (95%CI)	Begg test	Egger test
PFS	0.5 years	13	623	Random	0.781 (0.688-0.862)	0.3592	0.2175
	1 year	13	623	Random	0.387 (0.293-0.486)	0.6688	0.5978
	1.5 years	11	551	Random	0.117 (0.076-0.165)	0.3487	0.3386
	2 years	5	313	Random	0.069 (0.005-0.182)	0.6242	0.4217
OS	0.5 years	12	621	Random	0.943 (0.902-0.975)	0.2857	0.1556
	1 year	12	621	Random	0.690 (0.585-0.786)	0.3716	0.6814
	1.5 years	12	621	Random	0.385 (0.246-0.533)	0.4485	0.3606
	2 years	9	480	Random	0.212 (0.117-0.324)	0.4631	0.6764
	3 years	5	246	Fixed	0.056 (0.028-0.091)	0.8005	0.6243

OS, overall survival; PFS, progression-free survival; CI, confidence interval.

### Subgroup Analysis Stratified by Chemo(Embolization) Technique

TACE was adopted in 10 studies (21, 30–33, 38–42), and HAIC were in 5 studies (25, 34–37), respectively. **Table 4** exhibited the outcomes using TACE+TKIs+ICIs and HAIC+TKIs+ICIs, respectively. Briefly, the rates of CR, ORR, and DCR were slightly higher in the HAIC+TKIs+ICIs group than those in the TACE+TKIs+ICIs group, but on the contrary, a mild increase was observed in the rates of conversion, PFS at 0.5 years and 1 year, and OS at 1 and 2 years. Of note, the pooled rates of AEs grading exceeding three for TACE+TKIs+ICIs and HAIC+TKIs+ICIs were 0.235 (0.166-0.311) and 0.183 (0.047-0.376), respectively (**Table 4**).

**Table 4:** Subgroup analysis stratified by TACE or HAIC in triple therapy.

Endpoints		TACE+TKIs+ICIs				HAIC+TKIs+ICIs			
		Included studies	Participants	Effect model	Proportion (95%CI)	Includedstudies	Participants	Effect model	Proportion (95%CI)
CR		10	489	Random	0.110 (0.057-0.175)	5	252	Random	0.160 (0.033-0.349)
PR		10	489	Fixed	0.439 (0.395-0.484)	5	252	Fixed	0.460 (0.398-0.523)
ORR		10	489	Random	0.579 (0.502-0.653)	5	252	Random	0.664 (0.464-0.839)
DCR		10	489	Random	0.868 (0.798-0.926)	5	252	Fixed	0.910 (0.856-0.954)
PFS	0.5 years	8	371	Random	0.802 (0.667-0.911)	5	252	Random	0.743 (0.624-0.846)
	1 year	8	371	Random	0.362 (0.224-0.512)	5	252	Random	0.424 (0.326-0.526)
	1.5 years	6	299	Fixed	0.123 (0.086-0.164)	5	252	Random	0.124 (0.036-0.249)
	2 years	3	202	Random	0.072 (0.012-0.169)	2	111	Random	0.074 (0.000-0.487)
OS	0.5 years	8	396	Random	0.949 (0.895-0.987)	4	225	Random	0.932 (0.849-0.986)
	1 year	8	396	Random	0.724 (0.570-0.855)	4	225	Random	0.627 (0.521-0.726)
	1.5 years	8	396	Random	0.488 (0.317-0.660)	4	225	Random	0.200 (0.067-0.376)
	2 years	8	396	Random	0.226 (0.119-0.355)	1	84	Fixed	0.119 (0.059-0.208)
	3 years	5	246	Fixed	0.056 (0.028-0.091)	–	–	–	–
≥G3 AE		10	489	Random	0.235 (0.166-0.311)	5	252	Random	0.183 (0.047-0.376)
Conversion rate		2	132	Random	0.389 (0.146-0.665)	2	96	Random	0.334 (0.004-0.819)

TACE, transarterial chemoembolization; HAIC, hepatic artery infusion chemotherapy; TKIs, tyrosine kinase inhibitors; ICIs, immune checkpoint inhibitors; OS, overall survival; PFS, progression-free survival; CR, complete response; PR, partial response; ORR, objective response rate; DCR, disease control rate; AE, adverse events.

### Subgroup Analysis of Control Studies

There were 7 studies incorporating the control group (21, 25, 33–35, 39, 40), and the control group was TACE+TKIs in three studies (21, 33, 40), TKIs+ICIs in three (34, 35, 39), and TKIs in one (25), respectively. Results showed that TACE/HAIC+TKIs+ICIs was superior to TKIs alone in all fields (**Table 5**), and similar advantage were also observed compared to TACE+TKIs and TKIs+ICIs (all P<0.05, **Table 5**). Of note, there was no significant difference between TACE/HAIC+TKIs+ICIs and TKIs+ICIs in terms of the CR rate (P>0.05, **Table 5**) using a fixed effect model, and increasing severe AEs were not observed in the triple combination regimens.

**Table 5:** Subgroup analysis stratified by the administration and regimes in control group.

Endpoints	Studies included	Participants	I^2^	Effect model	HR/OR	95%CI	P-value
TACE+TKIs+ICIs vs. TACE+TKIs							
CR	3	274	0%	Fixed	2.99	1.09-8.19	0.03
PR	3	274	0%	Fixed	1.94	1.17-3.24	0.01
ORR	3	274	0%	Fixed	2.41	1.47-3.96	<0.001
DCR	3	274	0%	Fixed	2.61	1.54-4.43	<0.001
PFS	2	132	88%	Random	0.23	0.06-0.86	0.03
OS	3	274	45%	Fixed	0.48	0.36-0.64	<0.001
≥G3 AE	3	274	0%	Fixed	1.60	0.89-2.88	0.120
Conversion rate	1	142	–	–	2.77	1.12-6.88	0.030
TACE/HAIC+TKIs+ICIs vs. TKIs+ICIs							
CR	3	348	0%	Fixed	1.68	0.71-3.94	0.240
PR	3	348	0%	Fixed	2.34	1.46-3.73	<0.001
ORR	3	348	0%	Fixed	2.60	1.64-4.13	<0.001
DCR	3	348	59%	Random	3.44	1.44-8.24	0.006
PFS	3	348	40%	Fixed	0.63	0.51-0.76	<0.001
OS	3	348	0%	Fixed	0.54	0.43-0.68	<0.001
≥G3 AE	3	348	0%	Fixed	1.22	0.59-2.49	0.590
HAIC+TKIs+ICIs vs. TKIs							
CR	1	157	–	–	29.54	1.70-513.60	0.020
PR	1	157	–	–	5.92	2.83-12.39	<0.001
ORR	1	157	–	–	10.73	5.03-22.91	<0.001
DCR	1	157	–	–	7.24	2.98-17.60	<0.001
PFS	1	157	–	–	0.61	0.43-0.87	0.006
OS	1	157	–	–	0.40	0.24-0.67	<0.001
≥G3 AE	1	157	–	–	1.24	0.44-3.48	0.690
Conversion rate	1	157	–	–	26.30	1.50-460.26	0.030


TACE, transarterial chemoembolization; HAIC, hepatic artery infusion chemotherapy; TKIs, tyrosine kinase inhibitors; OS, overall survival; PFS, progression-free survival; CR, complete response; PR, partial response; ORR, objective response rate; DCR, disease control rate; AE, adverse events; HR, hazard ratio; OR, odds ratio; CI, confidence interval.

### Adverse Events

The pooled rates for the treatment related AEs were depicted in **Table 6**. No fatal AEs were reported in all the included studies. The top three most common AEs were elevated ALT (rate=0.436, 95%CI=0.326-0.550), elevated AST (rate=0.427, 95%CI=0.309-0.548), and hypertension (rate=0.295, 95%CI=0.246-0.346), respectively. The severe AEs were rarely reported, and the top three most common severe adverse events were hypertension (rate=0.061, 95%CI=0.027-0.105), elevated AST (rate=0.052, 95%CI=0.013-0.109), and elevated ALT (rate=0.048, 95%CI=0.013-0.101), respectively.

**Table 6:** Treatment-related adverse events of triple therapy.

Events	All grade				Grade≥3			
	Included studies	Participants	Effect model	Proportion (95CI)	Included studies	Participants	Effect model	Proportion (95CI)
Elevated ALT	9	439	Random	0.436 (0.326-0.550)	9	425	Random	0.048 (0.013-0.101)
Elevated AST	8	355	Random	0.427 (0.309-0.548)	9	425	Random	0.052 (0.013-0.109)
Elevated TBil	11	513	Random	0.274 (0.171-0.389)	10	429	Fixed	0.021 (0.007-0.040)
Thrombocytopenia	7	351	Random	0.183 (0.069-0.331)	7	337	Fixed	0.039 (0.018-0.064)
Decreased appetite	10	495	Random	0.198 (0.123-0.285)	10	481	Fixed	0.007 (0.000-0.025)
Fatigue	10	513	Random	0.231 (0.141-0.334)	10	499	Random	0.013 (0.001-0.034)
Hypertension	12	604	Random	0.295 (0.246-0.346)	12	590	Random	0.061 (0.027-0.105)
Abdominal pain	9	460	Random	0.243 (0.165-0.330)	8	376	Fixed	0.015 (0.003-0.034)
Diarrhea	12	593	Random	0.160 (0.117-0.207)	12	579	Fixed	0.017 (0.006-0.032)
Proteinuria	11	520	Random	0.204 (0.142-0.274)	11	520	Fixed	0.016 (0.005-0.032)
Hand–foot syndrome	10	495	Random	0.232 (0.162-0.309)	10	495	Fixed	0.029 (0.012-0.051)
Rash	10	503	Fixed	0.115 (0.087-0.145)	11	511	Random	0.013 (0.000-0.047)
Hypothyroidism	11	565	Random	0.165 (0.090-0.256)	12	573	Fixed	0.004 (0.000-0.015)
Fever	6	273	Random	0.312 (0.185-0.455)	6	259	Fixed	0.014 (0.000-0.038)
Nausea and vomiting	9	432	Random	0.157 (0.086-0.243)	9	418	Random	0.022 (0.001-0.060)
Arthralgia	4	119	Fixed	0.085 (0.038-0.146)	5	189	Fixed	0.004 (0.000-0.025)
Hoarseness	4	174	Fixed	0.060 (0.026-0.103)	4	174	Fixed	0.000 (0.000-0.016)
GI bleeding	4	213	Fixed	0.039 (0.014-0.072)	4	213	Random	0.008 (0.000-0.043)
RCCEP	4	194	Random	0.226 (0.103-0.379)	3	114	–	0.000
Mouth ulcers	3	158	Fixed	0.054 (0.021-0.098)	3	158	–	0.000

ALT, alanine aminotransferase; AST, aspartate aminotransferase; GI, gastrointestinal tract; RCCEP, reactive cutaneous capillary endothelial proliferation.

## Discussion

In the era of systemic therapy for intermediate–advanced HCC, is there still a niche for locoregional treatment including TACE and HAIC? In this systematic review, 741 patients in the 15 studies received the triple combination of TACE/HAIC, TKIs, and ICIs. Results showed that the triple combination provided a substantial CR rate of 0.124 (0.069-0.190), ORR of 0.606 (0.528-0.682), DCR of 0.885 (0.835-0.927), and prolonged PFS and OS without increased severe AEs. Further meta-analysis of control studies exhibited the superiority of the triple combination modality to TACE+TKIs, TKIs+ICIs, and TKIs alone.

Triple combination modality was firstly reported by Liu et al. (31) in 2021. A total of 22 patients with advanced HCC received TACE plus lenvatinib and camrelizumab, and at the first month, the ORR reached as high as 68.2%. The median PFS and OS were 11.4 and 24 months, respectively, without severe AEs during the treatment. From then on, more studies have been published with promising results. In this systematic review, 15 studies were identified with 741 patients receiving TACE/HAIC+TKIs+ICIs, and initial analysis showed encouraging results. However, the CR rate ranged from 0.069 to 0.190, the ORR ranged from 0.528 to 0.682, and the DCR ranged from 0.835 to 0.927, as well as the mean PFS (4.0-16.3 months) and mean OS (8.6-24.8 months). The divergences between the included studies might be attributed to the following reasons: 1) the sample size of all eligible studies was small, which meant that II error is hard to avoid; 2) there is substantial heterogeneity among uHCC patients, not to mention primary or recurrent HCC; 3) the regimen of triple therapy was very different from each other, including transarterial therapy modality (conventional TACE, DEB-TACE, or HAIC), TKI agents (sorafenib, Lenvatinib, or apatinib), and ICI agents (pembrolizumab, camrelizumab, tislelizumab, sintilimab, toripalimab, or nivolumab); 4) triple therapy as first-line treatment or not, which greatly influenced on tumor responses, median PFS and OS; 5) different treatment goals, for example, successful conversion and subsequent curative resection would have better prognosis than those with palliative treatment. Hence, the conclusion needs further validation, and **Table 7** exhibited ongoing prospective trials evaluating the clinical efficacy of the triple combination modality.

**Table 7:** Ongoing clinical trials for triple therapy.

Study design	Experimental arm	Control arm	Disease stage	Primary endpoint	Clinical trials, government registration
Phase 2	HAIC+TKIs+camrelizumab	None	Unresectable HCC	PFS	NCT05135364
Phase 1/ Phase 2	TACE+lenvatinib+sintilimab/camrelizumab	None	Advanced unresectable HCC	Conversion resection rate	NCT04997850
Phase 2	cTACE/DEB-TACE + FOLFOX regimen HAIC)+camrelizumab+apatinib	None	Advanced HCC	PFS	NCT04479527
Phase 3	TACE+lenvatinib+pembrolizumab	Oral placebo +IV Placebo +TACE	Incurable/Non-metastatic HCC	PFS/OS	NCT04246177
Phase 1	TACE+lenvatinib+ICIs	None	Intermediate/advanced HCC	Conversion resection rate	NCT04974281
Phase 2	TACE-HAIC+lenvatinib+ICIs	None	Intermediate/advanced HCC, without EHM	Conversion resection rate	NCT04814043
Phase 2	TACE+donafenib+ICIs	None	Advanced HCC	PFS	NCT05262959
Phase 2	TACE+sorafenib+ICIs	None	Intermediate/advanced HCC	ORR/OS	NCT04518852
Phase 2	TACE +sorafenib+tilelizumab	None	Advanced HCC	1-year survival rate	NCT04992143
Retrospective Observational	TACE+TKIs+ICIs	None	Intermediate HCC	OS	NCT05278195
Prospective Observational	cTACE/DEB-TACE-HAIC+ regorafenib+ICIs	cTACE/DEB- TACE-HAIC +regorafenib	Unresected HCC	ORR/PFS/OS	NCT05025592
Prospective Observational	HAIC+lenvatinib+sintilimab	None	HCC with PVTT	PFS	NCT04618367

HCC, hepatocellular carcinoma; cTACE, conventional transarterial chemoembolization; DEB-TACE, drug-eluting beads transarterial chemoembolization; HAIC, hepatic artery infusion chemotherapy; TKIs, tyrosine kinase inhibitors; ICIs, immune checkpoint inhibitors; PFS, progression-free survival; OS, overall survival; ORR, objective response rate; EHM, extrahepatic metastasis; PVTT, portal vein tumor thrombus.

Conversion therapy is well concerned nowadays in the field of uHCC (43, 44). Evidence suggests that R0 resection is a crucial independent protective factor of long-term survival (45). Shindoh et al. (46) found that advanced HCC patients after conversion therapy receiving R0 resection could have comparable prognosis with initially resectable HCC patients. Previous studies found that the successful conversion rate was 42.4% by lenvatinib and ICIs (47), 14.8% by TACE+sorafenib (48), and 12.8%-14.3% by HAIC+sorafenib (24, 49), respectively. Using the triple combination modality of HAIC+TKIs+ICIs, the conversion rate was reported to be as high as 60% by Zhang et al. (36), and in this systematic review, the pooled rate for the conversion surgery was 35.9%. The underlying mechanism of the synergistic effect of the triple combination might be as follows: 1) TACE or HAIC could improve the tumor immune microenvironment, induce continuous exposure to tumor antigens caused by continuous drug penetration, and thus enhance the efficacy of systemic therapies and 2) the anti-tumor angiogenesis effect of TKIs and ICIs will help eliminate tumor angiogenesis and tumor recurrence followed by TACE/HAIC, but both of them lack of validation in practice. Yang et al. (20) firstly identified that the triple therapy could not only activate cell immunity but also stimulate humoral immunity, and circulating Ig G, Ig λ, and Ig κ could serve as potential biomarkers of triple therapy. In the future, more attention should be paid on the triple combination modality for uHCC, especially for those with a strong willingness to receive radical resection.

It has been yet to be known which is the optimal modality of transarterial chemo(embolization) because there are rare reports comparing TACE, DEB-ATCE, and HAIC in the triple therapy for unresectable HCC. TACE has always been the cornerstone for intermediate-stage HCC (6), which was repeatedly confirmed by a recent systematic review with a median OS of 19.4 months (50). However, repeated TACE may lead to liver function impairment and even TACE resistance, and TACE alone is unsatisfactory for patients in advanced stage, especially portal vein invasion or extrahepatic spread (9, 10). Drug-eluting beads TACE (DEB-TACE) was found to yield better tumor responses and a similar safety profile compared to conventional TACE (5). Ren et al. (51) firstly compared the efficacy of DEB-TACE combined with ICI versus conventional TACE combined with ICI for unresectable HCC. Results showed that DEB-TACE was a safe and well-tolerated treatment and produced better PFS and tumor response in patients with unresectable HCC than conventional TACE. On the other hand, HAIC has been identified to be non-inferior to TACE in local control and even had a weak advantage over TACE in long-term prognosis (9–11). In this systematic review, a slight advantage of HAIC+TKIs+ICIs over TACE+TKIs+ICIs was observed in CR, ORR, and DCR, but it did not translate into survival benefit in PFS and OS. In addition, an apparent inferiority of HAIC+TKIs+ICIs to TACE+TKIs+ICIs was also found in the conversion rate (33.4% vs. 38.9%). Hence, it remains controversial in the choice of conventional TACE or DEB-TACE or HAIC among the triple combination, and “head-to-head” prospective trials might be the answer in the future.

As a saying goes, one size does not fit for all, and not all unresectable HCCs will benefit from the triple therapy. Ju et al. (39) found that TACE+apatinib+camrelizumab provided a clinical benefit for the subgroups of age <65 years old, men, PS score of 1, Child–Pugh classification of B, liver cirrhosis, hepatitis B infection, and AFP >200 mg/ml (all P<0.05), and similar findings were observed in the study of Zheng et al. (33). Mei et al. (35) found that HAIC+lenvatinib+ICIs exhibited a clinical benefit in patients with large, multiple HCCs (all P<0.05), but it failed in those with main portal vein tumor thrombus or extrahepatic metastasis (P>0.05), which was confirmed in a study of HAIC+lenvatinib+toripalimab. Further, Chen et al. (21) revealed that increased survival benefits with TACE+TKIs+ICIs was associated with the PD-L1 CPS score. Hence, identifying the potential beneficiary of the triple combination modality is an urgent agenda.

Safety is a bottleneck of the triple combination modality. The most common AEs are impaired liver function, fever, and abdominal pain related to TACE/HAIC (9–11); hypertension; diarrhea; and hand–foot syndrome to TKIs (52, 53) and rash, fatigue, and pruritus to ICIs (14, 19), respectively. A combination of TACE/HAIC and TKIs often increases the AEs of hypertension, hand–foot syndrome, and diarrhea, and a combination of TKIs and ICIs increases the risk of fatigue, rash, and hypothyroidism (20, 25, 31). As for the triple combination modality, safety can never be overemphasized. In this systematic review, the most common AEs were still elevated ALT and/or AST and hypertension, as well as the most severe AEs, but no mortality caused by the triple combination modality was reported. These results indicated that liver function might be selection criteria for the triple modality, and patients with impaired liver function will be contradicted to this modality.

Generally, the triple combination of TACE/HAIC, TKIs, and ICIs for uHCC needs a long way to go. Apart from the triple modality itself including the scheme and sequence, more factors should be of concern: 1) how many additional survival benefit, 2) how much cost-effectiveness, and 3) how about AEs with an intensified regimen. In addition, the accessibility of the medical care is another decision-making factor. In China, TKIs like sorafenib and lenvatinib and ICIs like camrelizumab and sintilimab have been enrolled into a healthcare insurance, which are much cheaper than nivolumab, pembrolizumab, and atezolizumab. Furthermore, TACE is much more preferred than HAIC, owing to its high compliance. Hence, more factors should be taken into consideration in the future trials.

There were several limitations in this systematic review. First, all of the studies were retrospective, in which recalling bias was hard to avoid. Second, considering that all the published studies came from China, the conclusion would not be applicable for the western patients due to the apparent heterogeneity in etiology between the East and the West. Third, data on the TACE/HAIC, TKIs, and ICIs were not available in several included studies; hence, the corresponding subgroup analysis could not be conducted. Fourth, considering that some studies came from the same center, the patient’s cohort might be the presence of overlap. Last but not the least, the sequential order of the triple modality was not unified among the included studies, and in the future, an extensive consensus should be reached on this issue.

## Conclusion

With the current data, we concluded that the triple combination of TACE/HAIC, TKIs, and ICIs would provide a clinical benefit for uHCC both in short- and long-term outcomes without increasing severe AEs. However, more is unknown on the optimal regimen, potential beneficiary, and latent AEs. Future RCTs with a larger sample size and cross-regional centers will aid in better clarifying the role of the triple modality for uHCC.

## Data Availability Statement

The original contributions presented in the study are included in the article/**Supplementary Material**. Further inquiries can be directed to the corresponding authors.

## Author Contributions

QK, FX, and HF: acquisition of data, analysis and interpretation of data. QK and LW: conception and design of the study. QK and LW: drafting the article. YZ and JL: critical revision, final approval. All authors contributed to the article and approved the submitted version.

## Funding

This work was supported by Fujian Provincial Clinical Research Center for Hepatobiliary and Pancreatic Tumors, Fujian, P.R.C. (2020Y2013), the Key Clinical Specialty Discipline Construction Program of Fuzhou, Fujian, P.R.C. (201912002), and the Startup Fund for scientific research, Fujian Medical University, Fujian, P.R.C. (2020QH1242).

## Conflict of Interest

The authors declare that the research was conducted in the absence of any commercial or financial relationships that could be construed as a potential conflict of interest.

## Publisher’s Note

All claims expressed in this article are solely those of the authors and do not necessarily represent those of their affiliated organizations, or those of the publisher, the editors and the reviewers. Any product that may be evaluated in this article, or claim that may be made by its manufacturer, is not guaranteed or endorsed by the publisher.

## Supplementary Material

Supplementary Figure 1 Funnel plot of complete response

Supplementary Figure 2 Sensitivity analysis of complete response

Supplementary Figure 3 Funnel plot of objective response rate

Supplementary Figure 4 Sensitivity analysis of objective response rate

Supplementary Figure 5 Funnel plot of disease control rate

Supplementary Figure 6 Sensitivity analysis of disease control rate
