# Corrigendum: The Significance of Transarterial Chemo(embolization) Combined With Tyrosine Kinase Inhibitors and Immune Check Point Inhibitors for Unresectable Hepatocellular Carcinoma in the Era of Systemic Therapy: A Systematic Review

**DOI:** 10.3389/fimmu.2022.952446

**Published:** 2022-06-07

**Authors:** Qiao Ke, Fuli Xin, Huipeng Fang, Yongyi Zeng, Lei Wang, Jingfeng Liu

**Affiliations:** ^1^ Department of Hepatopancreatobiliary Surgery, Mengchao Hepatobiliary Hospital of Fujian Medical University, Fuzhou, Fujian, China; ^2^ College of Clinical Medicine for Oncology, Fujian Medical University, Fuzhou, Fujian, China; ^3^ Department of Radiation Oncology, Fujian Cancer Hospital, Fujian Medical University Cancer Hospital, Fuzhou, Fujian, China; ^4^ Department of Hepatopancreatobiliary Surgery, Fujian Cancer Hospital, Fujian Medical University Cancer Hospital, Fuzhou, Fujian, China; ^5^ The United Innovation of Mengchao Hepatobiliary Technology Key Laboratory of Fujian Province, Mengchao Hepatobiliary Hospital of Fujian Medical University, Fuzhou, Fujian, China

**Keywords:** hepatocellular carcinoma, transarterial chemoembolization, hepatic arterial infusion chemotherapy, tyrosine kinase inhibitors, immune checkpoint inhibitors, systematic review

In the original article, references 47 and 48 was incorrectly inserted as

**47:** Wang YY, Wang LJ, Xu D, Liu M, Wang HW, Wang K, et al. Postoperative adjuvant transcatheter arterial chemoembolization should be considered selectively in patients who have hepatocellular carcinoma with microvascular invasion. *HPB (Oxford)* (2019) 21(4):425-33. doi: 10.1016/j.hpb.2018.08.001;

**48**: Wang L, Ke Q, Deng M, Huang X, Zeng J, Liu H, et al. Adjuvant transarterial chemoembolization for patients with hepatocellular carcinoma after radical hepatectomy: a real world study. *Scand J Gastroenterol* (2019) 54(11):1403-11. doi: 10.1080/00365521.2019.1684986**”**.

The correct references are as follows:

**47:** Zhang WW, Hu BY, Han J, Wang HG, Wang ZB, Ye HY, et al. A Real-World Study of PD-1 Inhibitors Combined With TKIs for HCC With Major Vascular Invasion as the Conversion Therapy: A Prospective, Non-Randomized, Open-Label Cohort Study. *Ann Oncol* (2020) 31:S1307. doi: 10.1016/j.annonc.2020.10.195;

**48:** Li CJ, Zhou J. Initial Report of a Two-Stage Resection for Hepatocellular Carcinoma Following Downstaging With Transarterial Chemoembolization and Sorafenib. *Fubu Waike* (2017) 30:295-8+301. doi: 10.3969/j.issn.1003-5591.2017.04.015.

The authors apologize for this error and state that this does not change the scientific conclusions of the article in any way. The original article has been updated.

## Publisher’s Note

All claims expressed in this article are solely those of the authors and do not necessarily represent those of their affiliated organizations, or those of the publisher, the editors and the reviewers. Any product that may be evaluated in this article, or claim that may be made by its manufacturer, is not guaranteed or endorsed by the publisher.

