# Supplementary figures and images for: The Significance of Transarterial Chemo(Embolization) Combined With Tyrosine Kinase Inhibitors and Immune Checkpoint Inhibitors for Unresectable Hepatocellular Carcinoma in the Era of Systemic Therapy: A Systematic Review
Source: Front Immunol. 2022 May 23;13:913464. doi: 10.3389/fimmu.2022.913464 (PMC9167927; doi:10.3389/fimmu.2022.913464)

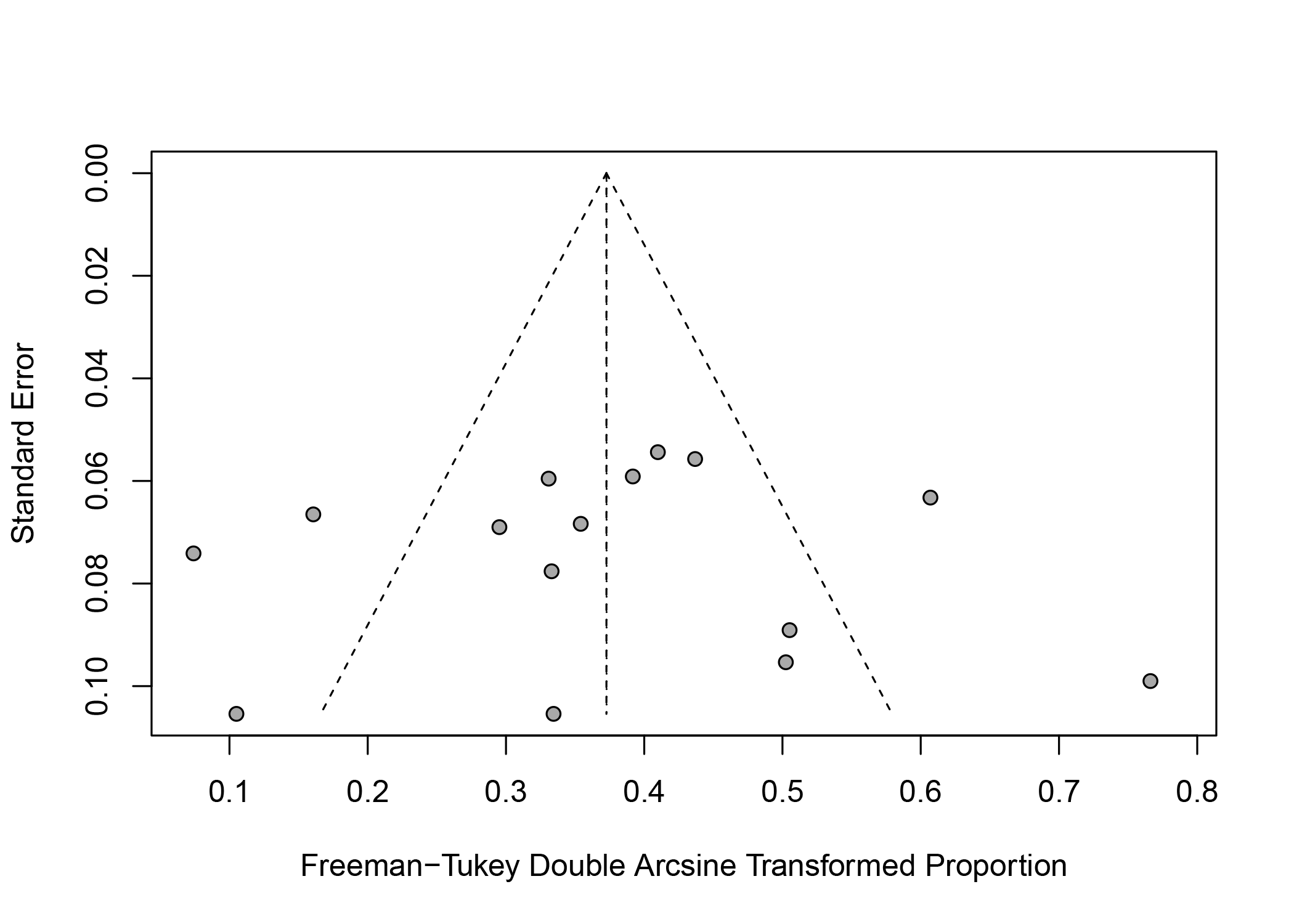

Supplement: Supplementary Figure 1 — Funnel plot of complete response [file Image_1.tif]

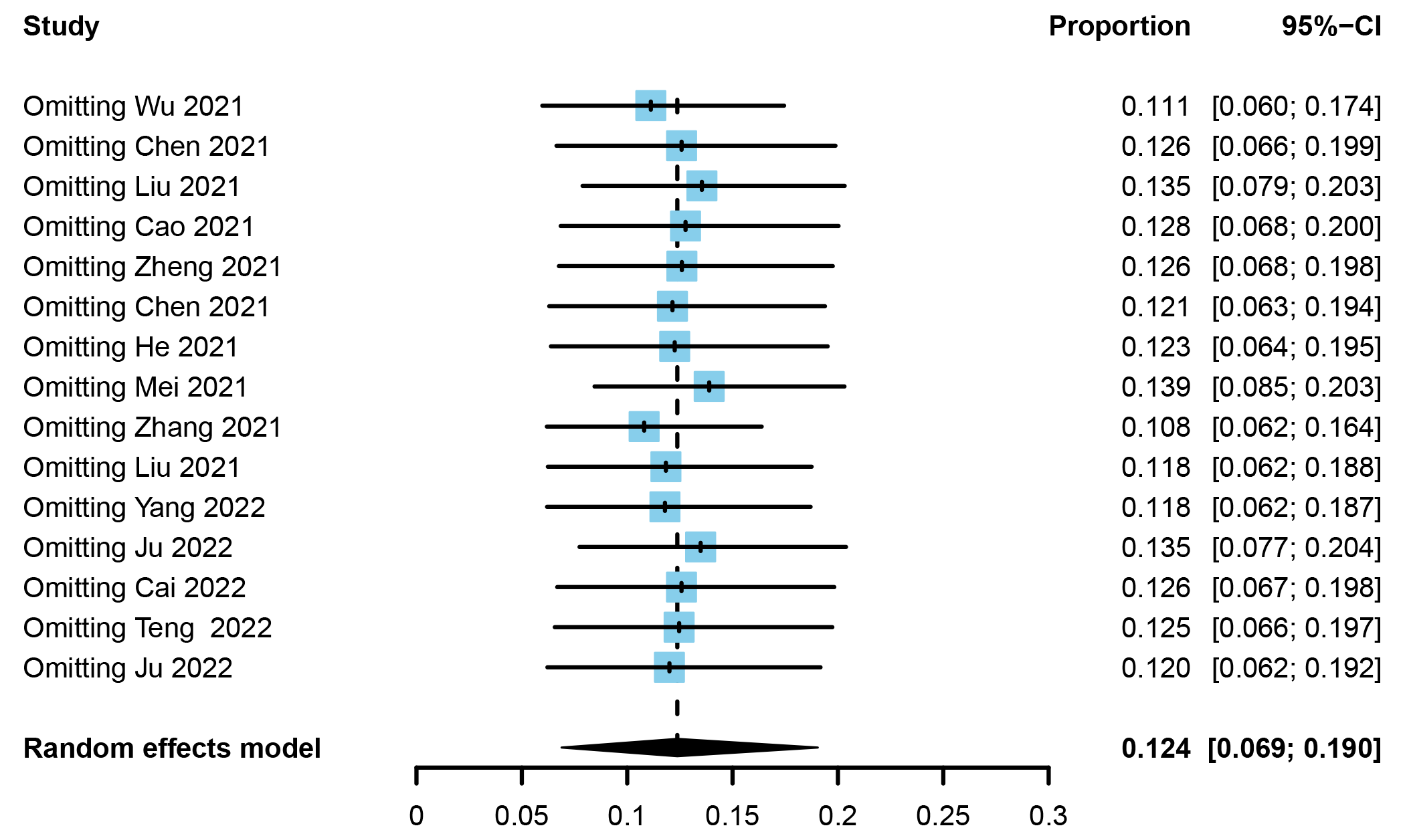

Supplement: Supplementary Figure 2 — Sensitivity analysis of complete response [file Image_2.tif]

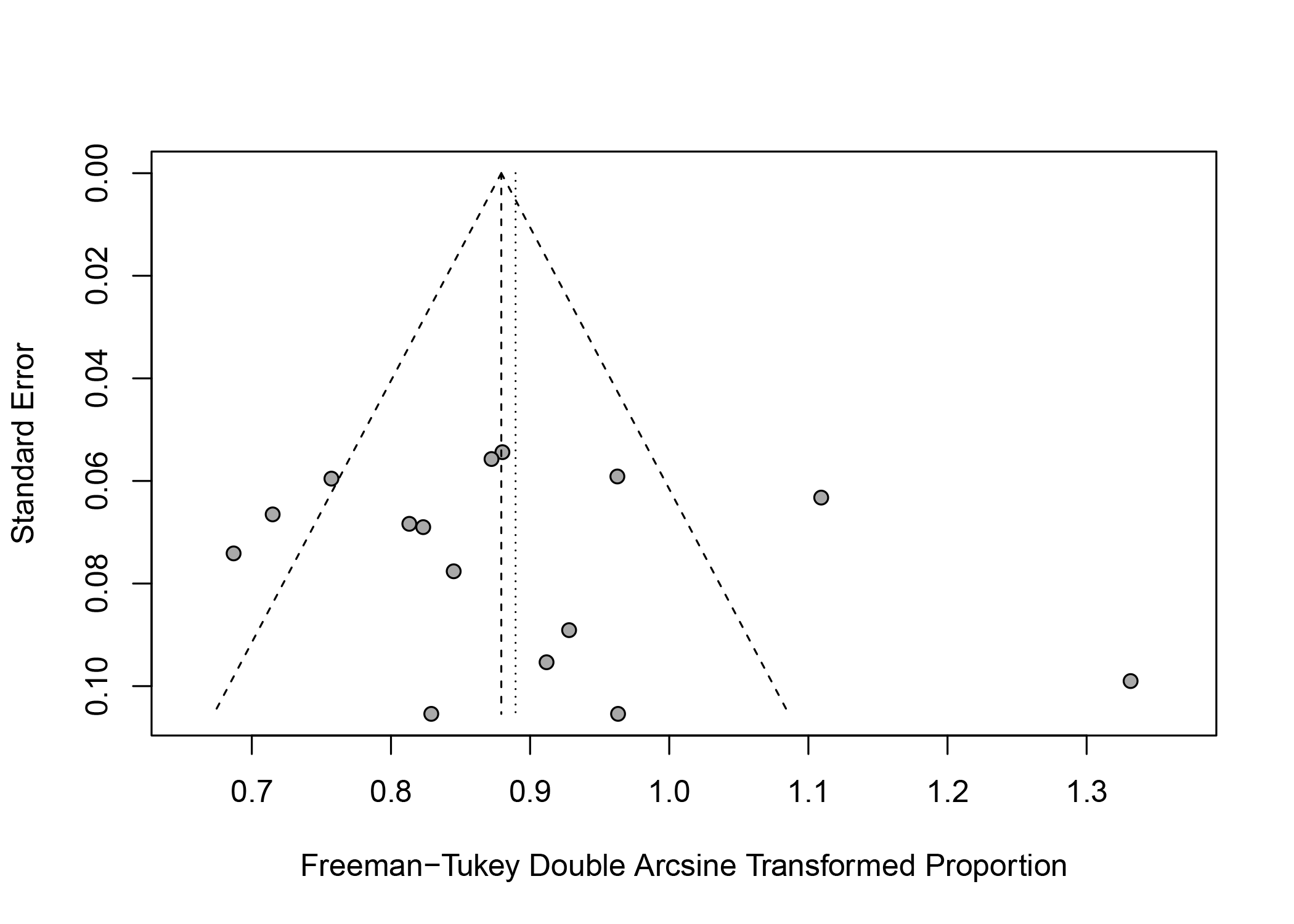

Supplement: Supplementary Figure 3 — Funnel plot of objective response rate [file Image_3.tif]

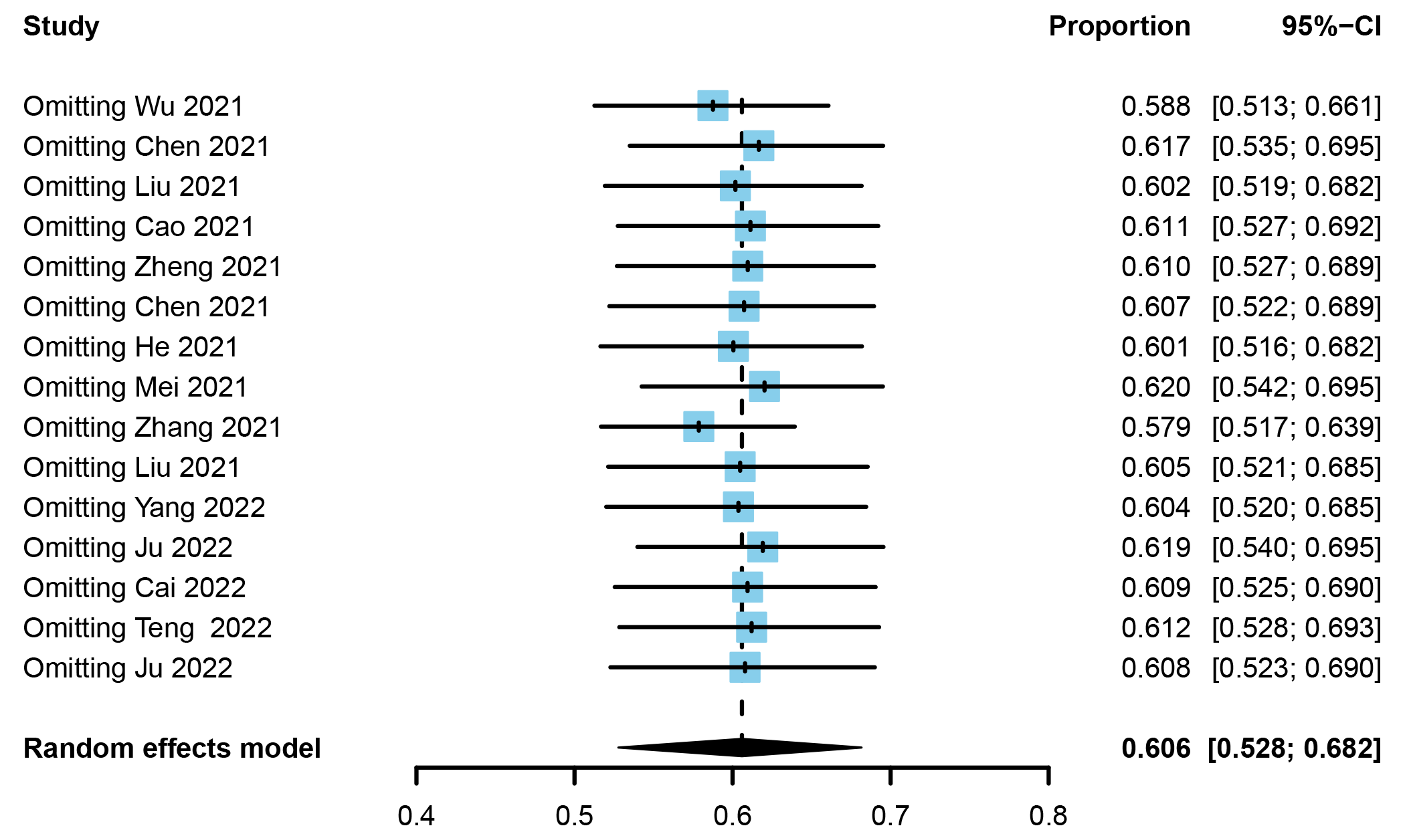

Supplement: Supplementary Figure 4 — Sensitivity analysis of objective response rate [file Image_4.tif]

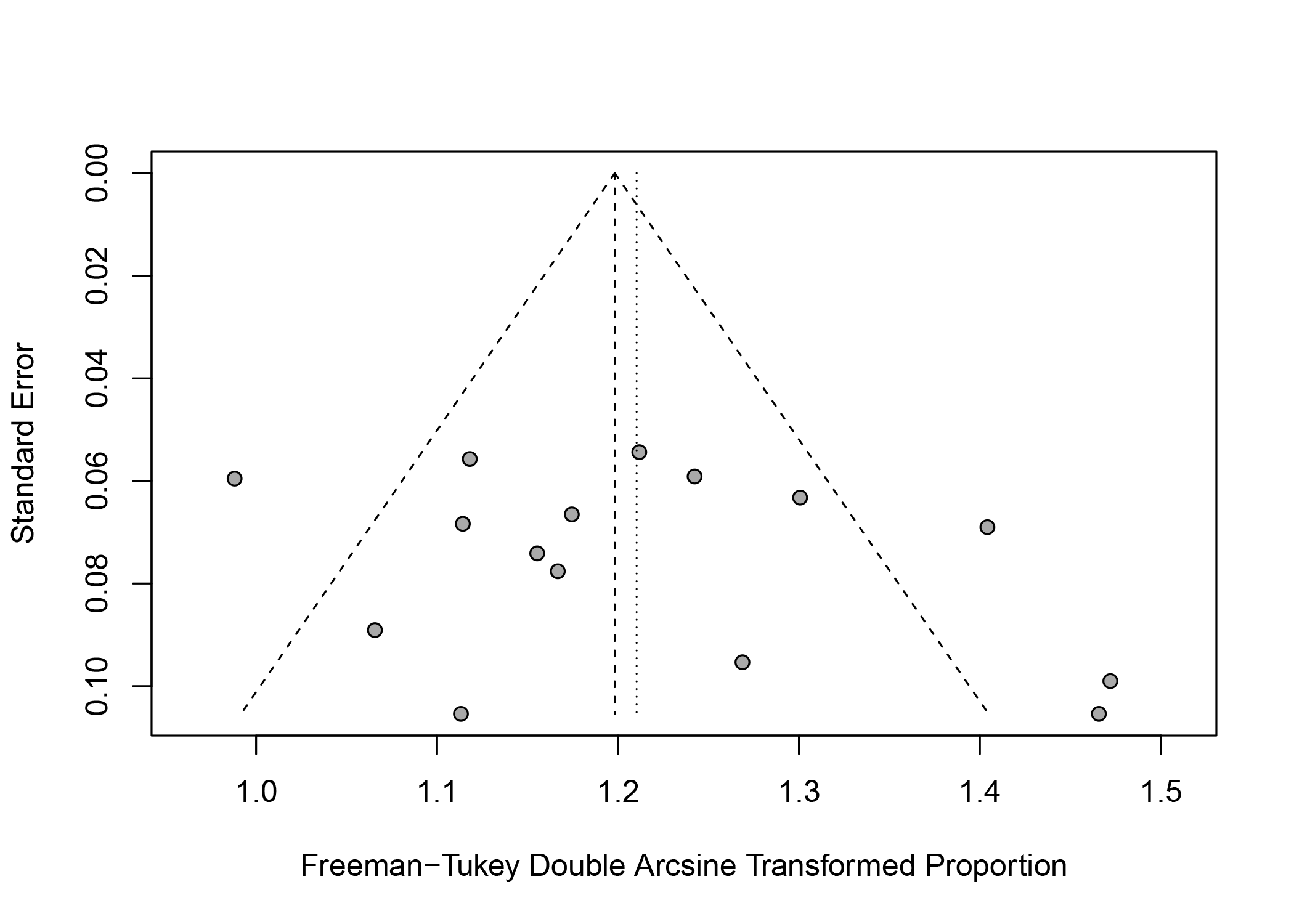

Supplement: Supplementary Figure 5 — Funnel plot of disease control rate [file Image_5.tif]

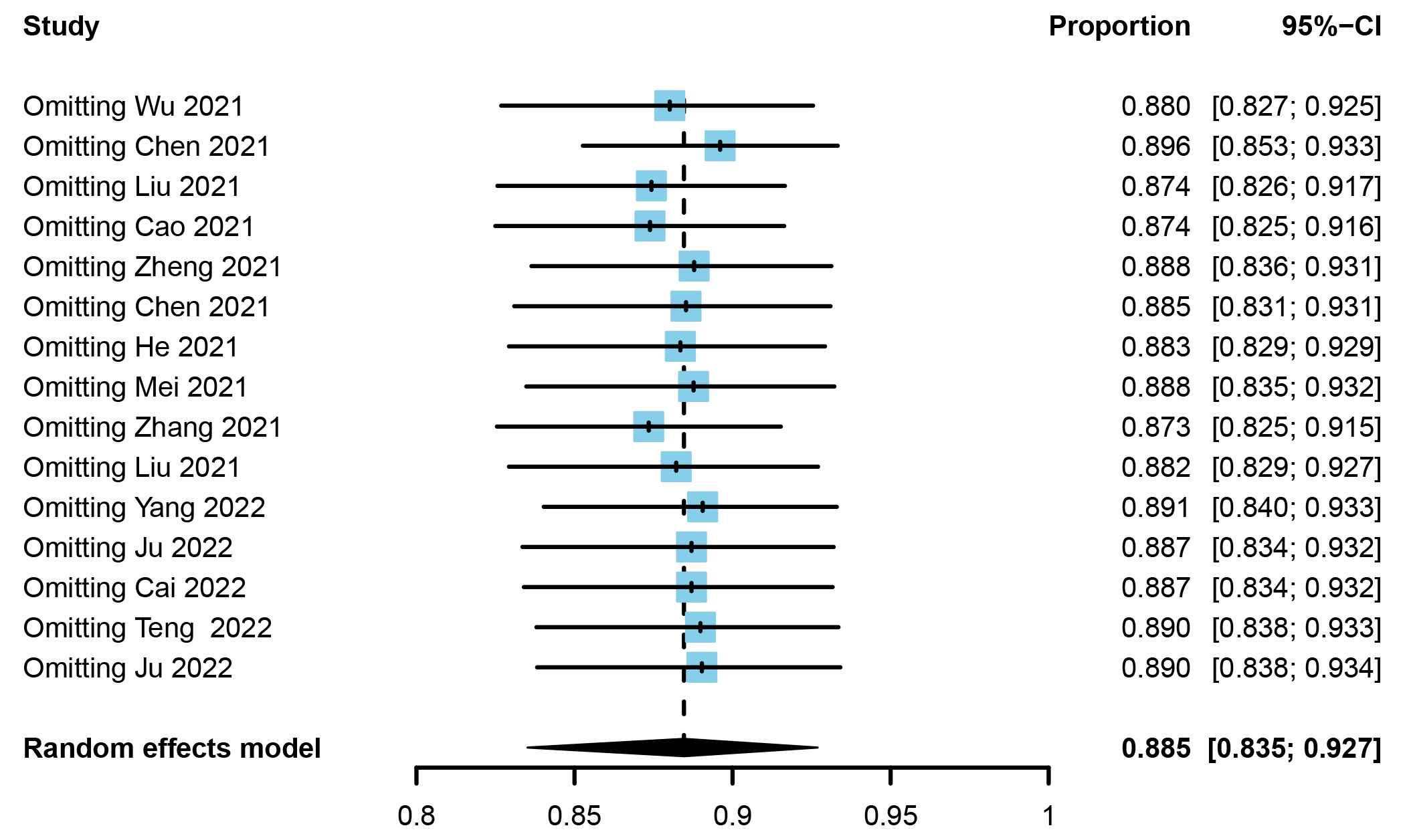

Supplement: Supplementary Figure 6 — Sensitivity analysis of disease control rate [file Image_6.tif]
